# First diagnosis of multisystem inflammatory syndrome in children (MIS-C): an analysis of PoCUS findings in the ED

**DOI:** 10.1186/s13089-021-00243-5

**Published:** 2021-09-08

**Authors:** Angelo G. Delmonaco, Andrea Carpino, Irene Raffaldi, Giulia Pruccoli, Emanuela Garrone, Francesco Del Monte, Lorenzo Riboldi, Francesco Licciardi, Antonio F. Urbino, Emilia Parodi

**Affiliations:** 1 Department of Pediatric Emergency, Regina Margherita Children’s Hospital, A.O.U. Città Della Salute E Della Scienza Di Torino, Turin, Italy; 2 Postgraduate School of Pediatrics, University of Turin, Piazza Polonia 94, 10126 Turin, Italy; 3 Department of Pediatric and Public Health Sciences, Regina Margherita Children’s Hospital, A.O.U. Città Della Salute E Della Scienza Di Torino, University of Turin, Turin, Italy

**Keywords:** SARS-CoV-2 infection, MIS-C, Children, PoCUS, LUS

## Abstract

Children with multisystem inflammatory syndrome (MIS-C) tend to develop a clinical condition of fluid overload due both to contractile cardiac pump deficit and to endotheliitis with subsequent capillary leak syndrome. In this context, the ability of point-of-care ultrasound (PoCUS) to simultaneously explore multiple systems and detect polyserositis could promote adequate therapeutic management of fluid balance. We describe the PoCUS findings in a case-series of MIS-C patients admitted to the Emergency Department. At admission 10/11 patients showed satisfactory clinical condition without signs and symptoms suggestive for cardiovascular impairment/shock, but PoCUS showed pathological findings in 11/11 (100%). In particular, according to Rapid Ultrasound in SHock (RUSH) protocol, cardiac hypokinesis was detected in 5/11 (45%) and inferior vena cava dilatation in 3/11 (27%). Peritoneal fluid was reported in 6/11 cases (54%). Lung ultrasound (LUS) evaluation revealed an interstitial syndrome in 11/11 (100%), mainly localized in posterior basal lung segments. We suggest PoCUS as a useful tool in the first evaluation of children with suspected MIS-C for the initial therapeutic management and the following monitoring of possible cardiovascular deterioration.

## Introduction

Multisystem inflammatory syndrome (MIS-C) is a newly defined severe pediatric illness related to previous SARS-CoV-2 infection [1]. The clinical presentation is usually nonspecific and features of MIS-C overlap with those of many other pediatric conditions. Most children present to the Emergency Department (ED) with common symptoms, such as fever and abdominal pain, in some cases associated with Kawasaki-like signs [2]. Nevertheless, MIS-C is a severe disease and prominent cardiac involvement has been reported in a high proportion of patients affected, requiring an intensive level of care in more than 50% of patients [3, 4]. For this reason, the prompt recognition of potential cardiovascular instability in the ED is crucial for patient care.

The usefulness of point-of-care ultrasound (PoCUS) in assessing adult and pediatric shock has already been reported [5, 6]. During the recent pandemic, PoCUS performed by pediatricians in ED has also been demonstrated as a useful method to detect lung abnormalities in children with MIS-C and COVID-19 [7, 8].

Herein, we describe the PoCUS findings in a case-series of MIS-C patients at ED admission.

## Materials and methods

In the ED of our Tertiary Care Pediatric Hospital (Regina Margherita Children’s Hospital -OIRM-, Turin, Italy), bedside PoCUS is routinely performed in high-risk children by experienced pediatricians. Our internal standard PoCUS protocol is derived from Copetti protocol [9, 10] for lung evaluation and from Rapid Ultrasound in SHock (RUSH) protocol [6] for cardiac and abdominal evaluation. Lung evaluation was performed with a linear or curvilinear probe (5–10 MHz) placed perpendicular, oblique and parallel to the ribs in the anterior, lateral and posterior (lower and upper) thorax and sitting positions were used to scan the posterior thorax. For RUSH protocol a curvilinear (2–5 MHz) and a phased array probe (2–8 MHz) were used (MyLab Seven; Esaote, Genoa, Italy).

On the 10th of January 2021, we performed a retrospective analysis of charts of all patients admitted to our ED with symptoms consistent with a presumptive diagnosis of MIS-C and subsequently discharged with a confirmed diagnosis between the 1st of April 2020 and the 31st of December 2020. The diagnosis of MIS-C at admission to the ED is presumptive. The current CDC case definition requires clinical, laboratory and anamnestic findings, without considering imaging features [1, 2].

Data of children who underwent PoCUS within the first 24 h from the admission were collected and retrospectively analyzed.

Archived PoCUS images were blind reviewed by three trained pediatricians with more than 5 years experience in bedside US, and then collegially discussed according to our internal protocol. Regarding lung, the presence of “pleural line irregularities”, “vertical artifacts”, “white lung” and “interstitial syndrome”, “consolidation” and “pleural effusion” was assessed. The interstitial syndrome is characterized by B-lines, arising from one point of the pleural line and from peripheral consolidations spreading down until the edge of the screen without fading [11, 12]. The pattern considered pathologic is the presence of multiple B-lines (at least three between two ribs in one longitudinal scan) fanning out from the lung–wall interface [9, 10, 13]. Regarding cardiac evaluation, we defined “cardiac kinetic alteration” the presence of an abnormal size or contractility status of the left ventricle and/or a relative increase of the size of right ventricle (i.e., hypocontractile hearth) compared to the left ventricle. Both B mode and M-Mode tracing were used to evaluate systolic fractional shortening, according to RUSH protocol [6]. The pericardial sac was visualized to assess the presence of “pericardial effusion”.

The evaluation of inferior caval vein positioning the probe in the epigastric area in a long-axis configuration was used in order to estimate the intravascular volume. A reduced compliance to respiratory cycles (i.e., IVC Sniff Test) was defined as “IVC dilatation”.

Data regarding reasons for ED admission, clinical features including signs and symptoms of cardiovascular involvement and/or shock (capillary refill time > 2 s, cold extremities, hypotension, shortness of breath, severe tachycardia), laboratory data and X-ray imaging findings were collected, too.

The statistical analysis was performed using IBM SPSS statistics package 27.0. A descriptive analysis of the variables was conducted. The data were reported as absolute numbers and percentages for categorical variables while as medians and range for continuous variables.

## Results

**Fig. 1:**
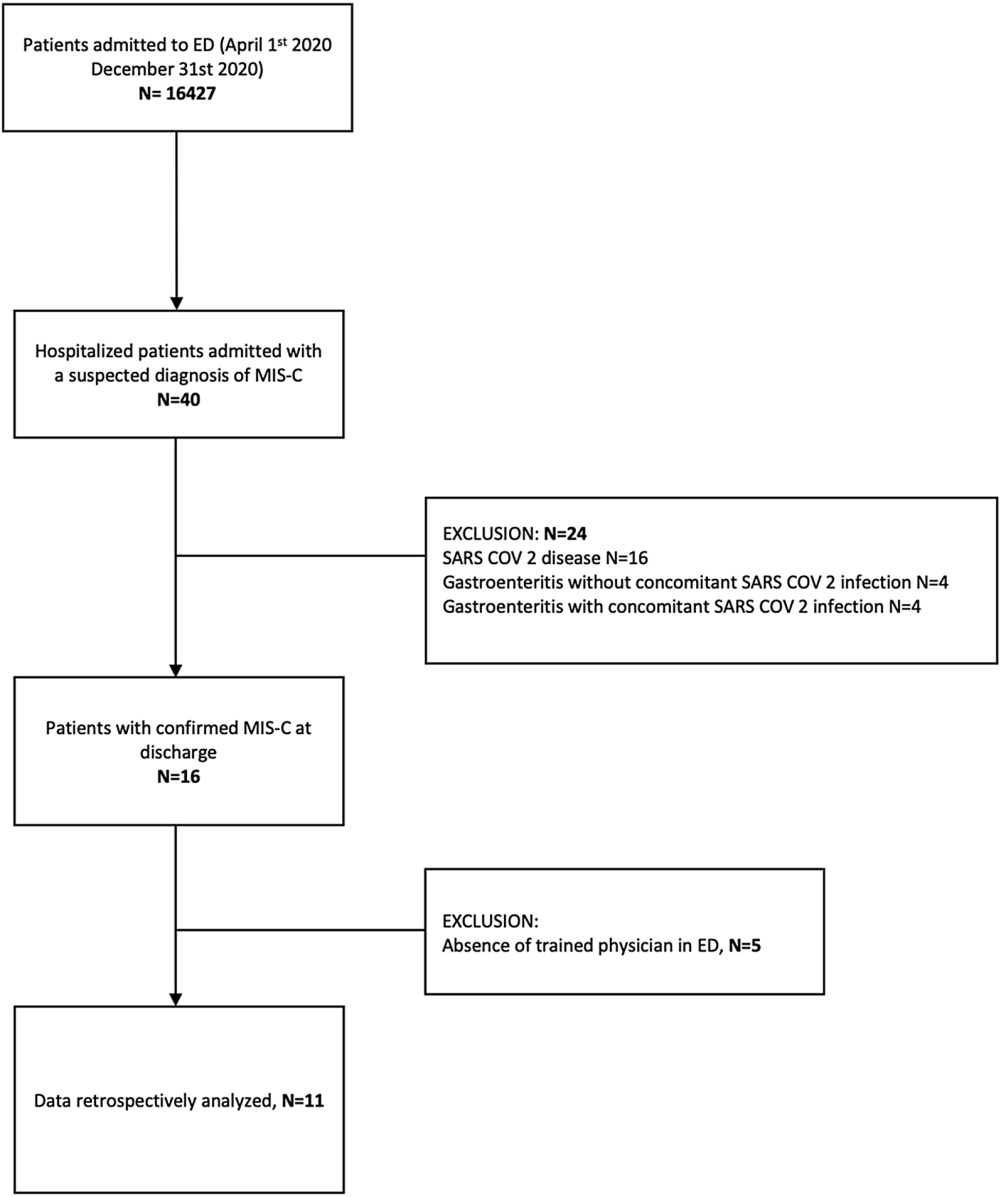
Flowchart of patient enrollment and exclusion criteria

Between the 1st of April and the 31th December 2020, 40 patients presented to our ED with a high suspicion of MIS-C disease with fever for more than 24 h and presence of laboratory evidence of inflammation or SARS-CoV-2 infection. Twenty-four/40 received alternative diagnosis during hospitalization. The remaining 16 were subsequently discharged with a confirmed diagnosis according to CDC case definition; of these, 11 (69%) underwent PoCUS in the first 24 h from admission in the ED and were enrolled in the study (see Fig. 1).

Eleven/11 (100%) presented to ED for fever, associated with abdominal pain in 8/11 (73%), vomiting in 6/11 (55%), and Kawasaki-like signs (i.e., mucositis and conjunctivitis) in 3/11 (27%). At admission 10/11 (91%) showed good clinical condition without signs and symptoms suggestive for cardiovascular impairment/shock, with the exception of the only child appearing in critical condition, presenting severe hypotension and requiring admission to intensive care unit. PoCUS showed pathological findings in 11/11 (100%). According to RUSH protocol, cardiac hypokinesis was detected in 5/11 (45%) and inferior vena cava dilatation in 3/11 (27%). Peritoneal fluid was reported in 6/11 cases (54%). Lung ultrasound (LUS) evaluation revealed an interstitial syndrome in 11/11 (100%), mainly localized in posterior basal lung segments. One child appearing in critical condition at admission showed alteration both at LUS and RUSH protocol (Table 1, patient number 3).

**Table 1:** PoCUS and X-rays findings, reason for ED admission and NT-pro BNP value at admission in children with MIS-C

	Patient 1	Patient 2	Patient 3	Patient 4	Patient 5	Patient 6	Patient 7	Patient 8	Patient 9	Patient 10	Patient 11
Reason for ED admission	Fever, abdominal pain	Fever, abdominal pain, vomiting and diarrhea	Fever, abdominal pain, vomiting	Fever, abdominal pain, vomiting, conjunctivitis	Fever, headache, abdominal pain, vomiting and diarrhea	Fever, abdominal pain, vomiting and diarrhea	Fever, abdominal pain, vomiting and diarrhea, rash, conjunctivitis	Fever, conjunctivitis	Fever, neck and scrotal pain, headache, fatigue	Fever, cough, abdominal pain, rash	Fever, headache, chest pain
NT-proBNP value (ng/L)	NA	NA	12,571	31,820	9211	557	976	1050	10,097	15,284	1452

PoCUS	All	Patient 1	Patient 2	Patient 3	Patient 4	Patient 5	Patient 6	Patient 7	Patient 8	Patient 9	Patient 10	Patient 11
Copetti LUS protocol												
Pleural line irregularities	11/11 (100%)	Bilateral	Bilateral	Bilateral	Bilateral	Bilatateral	Bilateral	Bilateral	Bilateral	Bilateral	Bilateral	Bilateral
Vertical artifacts	11/11 (100%)	Yes	Yes	Yes	Yes	Yes	Yes	Yes	Yes	Yes	Yes	Yes
White lung	2/11 (18%)	No	No	Yes	No	No	No	No	No	Yes	No	No
Consolidation	4/11 (36%)	No	No	Yes	Yes	Yes	No	No	No	Yes	No	No
Pleural effusion	6/11 (54%)	No	No	Yes	Yes	No	Yes	No	No	Yes	Yes	Yes
RUSH protocol												
Cardiac Kinetic alteration	5/11 (45%)	No	No	Yes	Yes	Yes	No	Yes	No	Yes	No	No
Pericardial effusion	3/11 (27%)	No	No	Yes	No	No	No	Yes	No	Yes	No	No
IVC dilatation	3/11 (27%)	No	No	Yes	No	Yes	No	No	No	Yes	No	No
Peritoneal fluid	6/11 (54%)	No	Yes	Yes	Yes	Yes	No	No	No	Yes	No	Yes
Chest X Ray		Normal	Normal	Perihilar interstitial thickening, cardiomegaly	Consolidation, perihilar interstitial thickening, cardiomegaly	Normal	NA	NA	Atelectasis	Normal	Normal	Pleural effusion, cardiomegaly

**Fig. 2:**
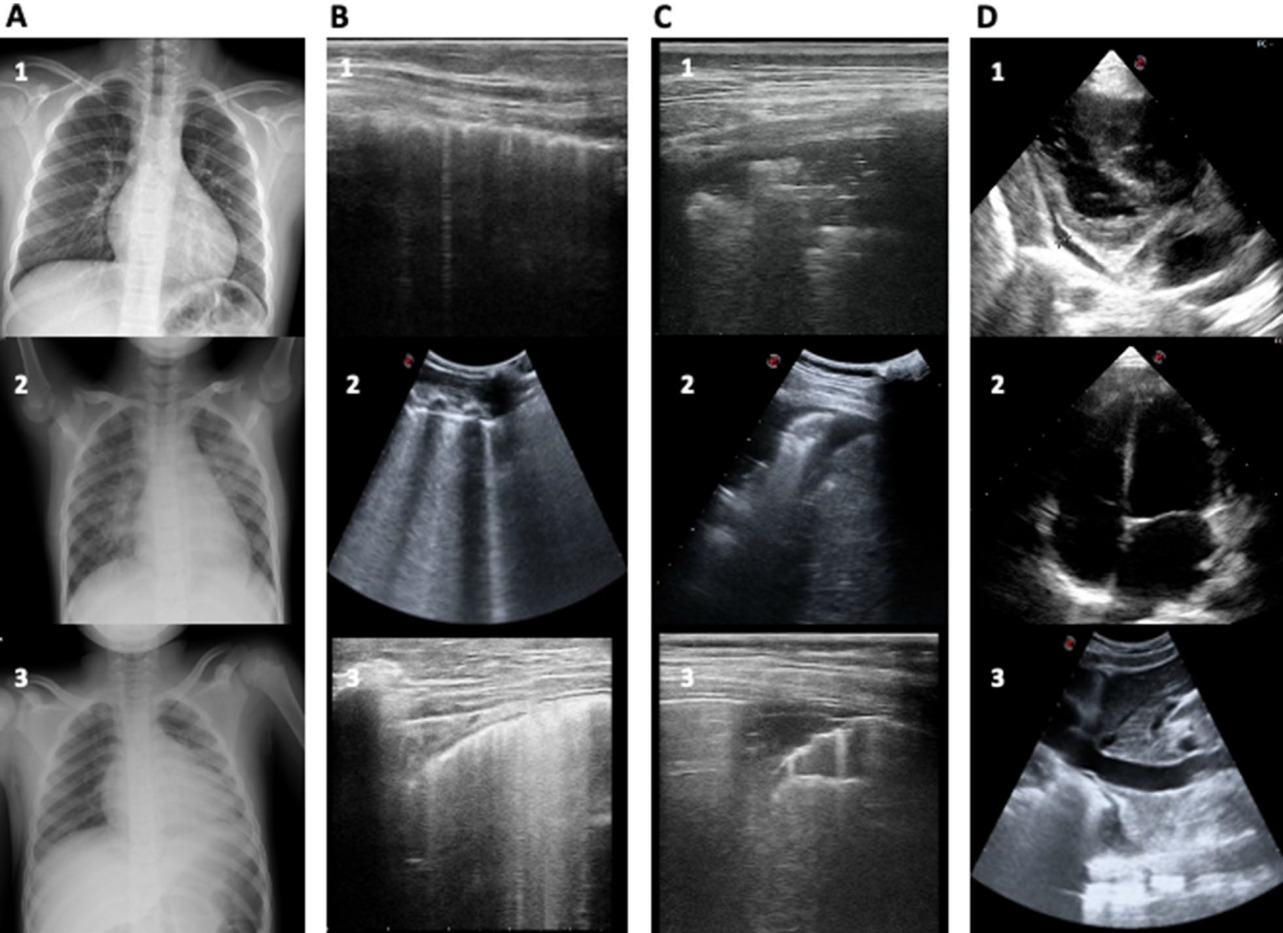
Imaging findings: **A** chest X-ray findings;** B** LUS exam: **B1** initial interstitial pattern, **B2** vertical artifacts, **B3** white lung; **C** LUS exam: **C1** lung consolidations; **C2**, **C3** lung consolidations with pleural effusion; **D** RUSH exam: **D1** pericardial effusion, **D2** dilated cardiac chambers, **D3** plethoric IVC

During hospitalization all children developed cardiac injury with a median NT-pro BNP level of more than 8500 ng/l and pathological findings at echocardiogram. Detailed PoCUS and chest X-ray findings, as well as the first detection of N-terminal pro B-type natriuretic peptide (NT-pro BNP), are reported in Table 1. The five patients (Patient Number 3,4,5,7 and 9, Table 1) with kinetic cardiac alteration displayed a hypocontractile heart confirmed by the ejection fraction measurement at subsequent echocardiogram. Peculiar imaging findings at chest X-ray, LUS and RUSH exam are shown in Fig. 2.

## Discussion

Even though the radiographic features of COVID-19 in children have been thoroughly reported [9], data describing imaging findings in MIS-C are limited. Recently, a prospective observational study summarizing chest and abdominal imaging findings has been published [14].

To the best of our knowledge, for the first time we report here a detailed analysis on the PoCUS findings in the ED patients with confirmed MIS-C [7].

In our case-series, PoCUS was performed in 11 children with MIS-C within 24 h from hospitalization. Most patients presented to the ED for fever and abdominal pain in good clinical condition. However, the development of cardiac injury in all children during hospitalization (median NT-pro BNP level of more than 8500 ng/l) is consistent with the already reported clinical instability of MIS-C patients, who are at risk for sudden cardiovascular worsening [4]. For this reason a strict clinical, laboratory and imaging monitoring is strongly recommended.

In our case-series, despite initial fair clinical condition, PoCUS revealed early cardiac hypokinesis in almost half of our patients and IVC dilatation in three [15]. As a consequence, PoCUS allowed early identification of cardiopulmonary involvement, even before the patients developed symptoms suggestive for cardiovascular impairment/shock.

The PoCUS findings also showed abdominal free fluid in more than half of our patients, confirming the frequent involvement of gastrointestinal tract previously described [2, 14].

Children with MIS-C tend to develop a clinical condition of fluid overload due both to contractile cardiac pump deficit and to endotheliitis with subsequent capillary leak syndrome. In this context, the ability of PoCUS to simultaneously explore multiple systems and detect polyserositis promotes adequate therapeutic management of fluid balance.

Finally, all our patients presented at least one alteration at LUS examination, with the predominance of the interstitial pattern, underlying an inflammatory and edemigenous genesis [16]. Furthermore, pathological findings in the X-ray were reported in only 3/11 cases (27%), suggesting its marginal role in the initial evaluation (Fig. 2) [17].

PoCUS has unquestionable advantages: the ability to explore at a first sight the multi-organ involvement typical of MIS-C; the possibility of being performed at any moment by a pediatrician in the ED; and the opportunity to be repeated without any risk during the follow-up.

We are aware of the limits of our study (i.e., the retrospective nature of the analysis and the small sample size). However, if our data will be confirmed by further research on larger samples, PoCUS might be considered as a useful and non-invasive tool in the first evaluation of children with suspected MIS-C, although a specific MIS-C-correlated ultrasound pattern has not been identified. Moreover, it could play a key role to help physicians also in the therapeutic management of fluid balance, as well as in the following monitoring of these pediatric patients, at risk for cardiovascular deterioration.

## Data Availability

Not applicable.

## Abbreviations

ED: Emergency department
IVC: Inferior vena cava
LUS: Lung US
MIS-C: Multisystem inflammatory syndrome in children
NT-pro BNP: N-terminal pro B-type natriuretic peptide
PoCUS: Point-of-care ultrasound
RUSH: Rapid Ultrasound in SHock
RT-PCR: Real-time polymerase chain reaction
US: Ultrasound
